# Influence of loneliness burden on cardio-cerebral vascular disease among the Chinese older adult: a national cohort study

**DOI:** 10.3389/fpubh.2024.1307927

**Published:** 2024-02-13

**Authors:** Dishan Wu, Xing Hu, Lingbing Meng, Jianyi Li, Jiapei Xu, Luyao Zhang, Qinan Ma, Hui Li, Xuezhai Zeng, Juan Li, Qiuxia Zhang, Deping Liu

**Affiliations:** ^1^Department of Cardiology, Beijing Hospital, National Center of Gerontology, Institute of Geriatric Medicine, Chinese Academy of Medical Sciences, Beijing, China; ^2^Graduate School, Chinese Academy of Medical Sciences and Peking Union Medical College, Beijing, China; ^3^Health Service Department of the Guard Bureau of the Joint Staff Department, Beijing, China; ^4^Graduate School, Peking University Fifth School of Clinical Medicine, Beijing, China; ^5^Center on Aging Psychology, Key Laboratory of Mental Health, Institute of Psychology, Chinese Academy of Sciences, Beijing, China; ^6^State Key Laboratory of Brain and Cognitive Science, Institute of Biophysics, Chinese Academy of Sciences, Beijing, China; ^7^China Research Center on Aging, Beijing, China

**Keywords:** loneliness, cardio-cerebral vascular diseases, cumulative burden, older adult, longitudinal change

## Abstract

**Background:**

Adverse psychosocial factors play an important role in cardio-cerebral vascular disease (CCVD). The aim of this study was to evaluate the impact of the cumulative burden of loneliness on the risk of CCVD in the Chinese older adult.

**Methods:**

A total of 6,181 Chinese older adult over the age of 62 in the monitoring survey of the fourth Sample Survey of the Aged Population in Urban and Rural China (SSAPUR) were included in this study. The loneliness cumulative burden (scored by cumulative degree) was weighted by the loneliness score for two consecutive years (2017–2018) and divided into low- and high-burden groups. The outcome was defined as the incidence of CCVD 1 year later (2018–2019). A multivariate logistic regression model was used to examine the relationship between the cumulative burden of loneliness and the new onset of CCVD.

**Results:**

Among participants, 18.9% had a higher cumulative burden of loneliness, and 11.5% had a CCVD incidence within 1 year. After multivariate adjustment, the risk of developing CCVD in the high-burden group was approximately 37% higher than that in the low-burden group (OR 1.373, 95%CI 1.096–1.721; *p* = 0.006). Similar results were obtained when calculating the burden based on cumulative time. Longitudinal change in loneliness was not significantly associated with an increased risk of CCVD. A higher cumulative burden of loneliness may predict a higher risk of developing CCVD in older adult individuals aged 62–72 years or in those with diabetes.

**Conclusion:**

The cumulative burden of loneliness can be used to assess the risk of new-onset CCVD in the older adult in the short term.

## Background

Cardio-cerebral vascular disease (CCVD) is the leading cause of death worldwide. According to the Global Burden of Disease Research Project (GBD) statistics, the number of cardiovascular deaths worldwide has increased by 12.5% in the past decade, accounting for approximately one-third of total deaths (1). In China, nearly 4 million people died of cardiovascular diseases every year by 2016 (2, 3). Aging has become a global development trend with changes in the world population structure and is also the main risk factor for CCVD (4, 5). The annual cardiovascular events in China are expected to increase by more than 50% from 2010 to 2030, and the mortality rate will also increase, mainly in people aged 65–84 years (6). There are many mechanisms of CCVD, among which psychosocial factors such as loneliness play an important role (7).

As age increased, loneliness became stronger (8). It was defined as a subjective state in which there was a gap between the actual social relationship and the expected value (9) and it was likely to be the vector of social isolation leading to depression or other diseases (10). In the middle-aged and older adult populations, there was a significant correlation between depression and cardiovascular events, among which loneliness, as one of the measurement indicators of depression, may play an essential role (11). However, a study of 479,054 people in the British biological database who were followed up for 7.1 years found that the increased mortality of participants with a history of acute myocardial infarction or stroke was related to social isolation but not loneliness (12). A prospective cohort study (13) found that loneliness increased with age in older adult men; however, there was no independent association between loneliness and the risk of all-cause, cardiovascular, or non-cardiovascular death. Considering that most previous studies used a single point in time or a single trend of change to assess loneliness for risk prediction, the effectiveness was weak, while the subjective emotions changed over time and were deficient in stability. Therefore, finding an indicator that can better represent the severity of loneliness is essential for studying its relationship with disease.

Currently, there is a lack of studies describing loneliness using cumulative burden, and the relationship between cumulative burden and short-term cardiovascular events is not clear. Our study aimed to explore the role of the cumulative burden of loneliness in assessing the risk of CCVD through a nationally representative longitudinal tracking dataset.

## Methods

### Study population and design

The fourth Sampling Survey of Aged Population in Urban and Rural China (2015) (SSAPUR, 2015) was conducted by the National Working Committee on Aging. The specifics of the survey have been detailed in previous research (14). The survey adopted a stratified and multistage complex sampling method. It surveyed the older adult aged 60 years and above from 1 August 2015, in 31 provinces, autonomous regions, and municipalities. The dimensions covered by the survey include demography, economy, health, spirituality, culture, social participation, livability, and many other aspects. This cohort was a follow-up survey that selected 10% of the 2015 survey sample (approximately 22,000 people) for continuous tracking.

**Figure 1:**
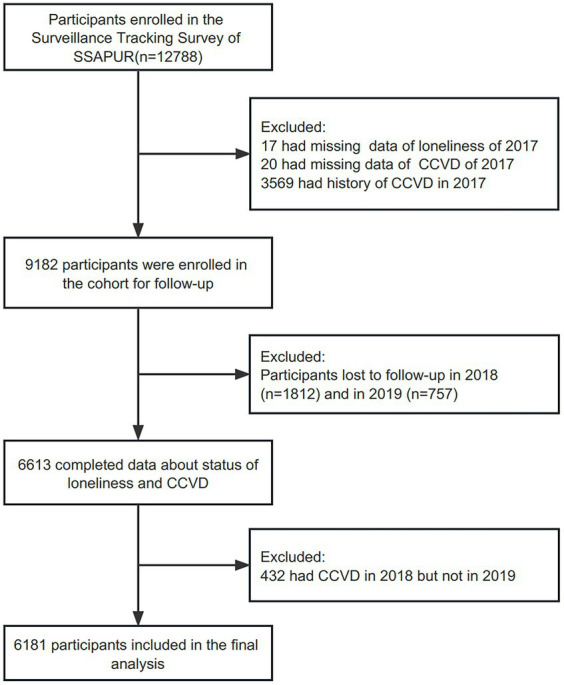
Flowchart of study.

The enrollment flow of this prospective cohort study is shown in Figure 1. A total of 12,788 participants were chosen for the follow-up survey in 2017, and 6,613 participants had completed data on loneliness and CCVD status at baseline. Among these, 3,569 subjects with a history of CCVD were excluded, and 2,562 participants were lost to follow-up. In addition, 432 participants who had CCVD in 2018 but not in 2019 were excluded. Finally, 6,181 subjects were included in this analysis.

This study was approved by the Ethics Review Committee of the Beijing Hospital (2021BJYYEC-294-01). All the participants provided written informed consent.

### Exposure

**Figure 2:**
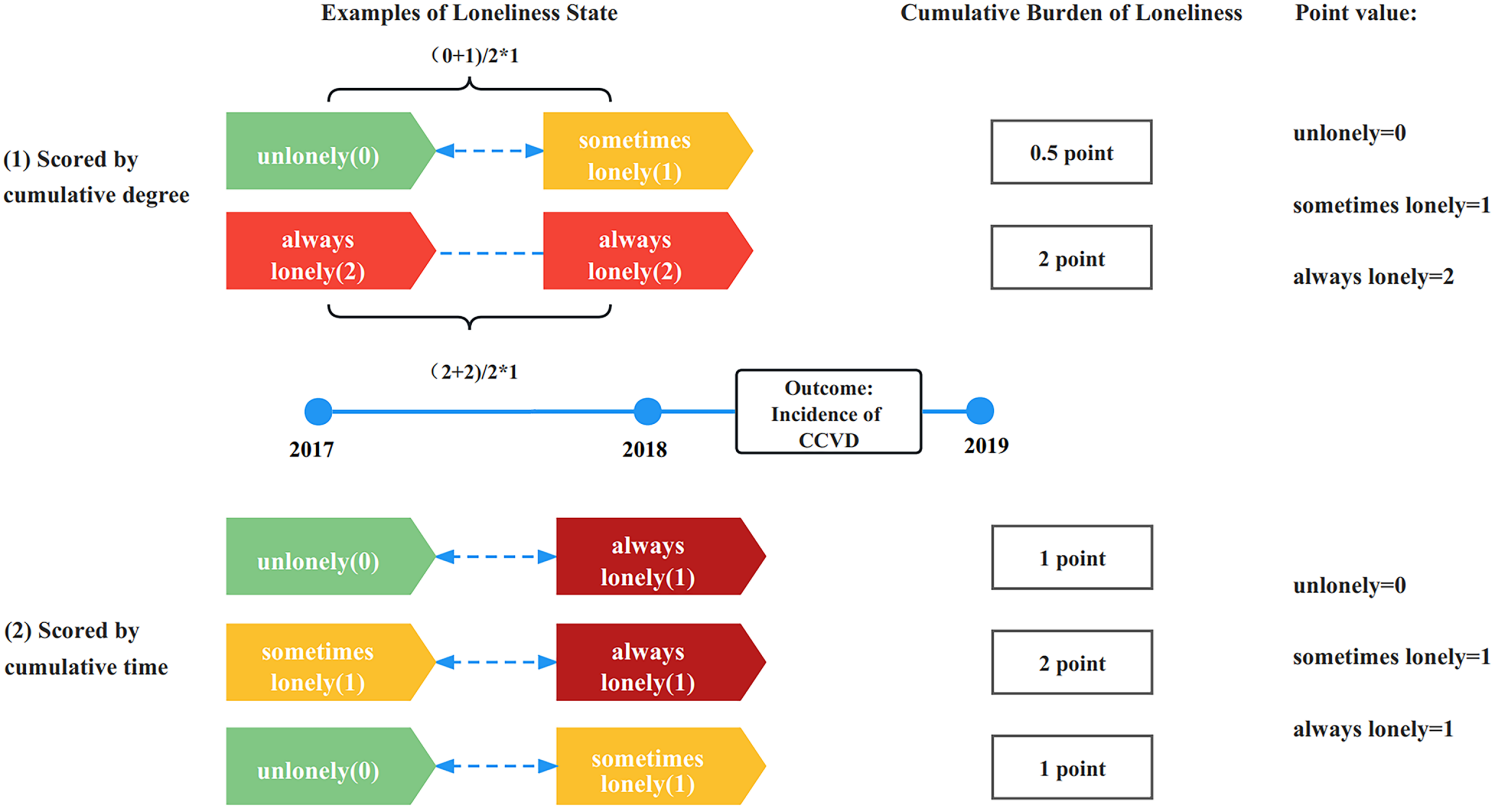
Schematic diagram of two different cumulative burden scores of loneliness.

Loneliness was measured through the questionnaire: Do you feel lonely? 1 usually, 2 sometimes, and 3 never (15). The answers were assigned according to the severity of loneliness from low to high: 2 points for usually feeling lonely, 1 point for sometimes feeling lonely, and 0 points for never feeling lonely. A higher score indicated greater loneliness (16). Two calculation methods for the cumulation burden of loneliness were designed in this study: (1) scored by cumulative degree: Calculated by the weighted average method according to the following formula: cumulative burden = ((value2017 + value2018)/2) × 1 years (17). The scores ranged from 0 to 2 and increased at 0.5-point intervals. The receiver operating characteristic curve was used to determine the optimal cut-off value, and the cumulative burden of loneliness was divided into low- and high-burden groups based on this value (0.75). (2) scored by cumulative time: In addition, the scores can be assigned a value of 0 or 1 according to whether they were exposed to loneliness each year (choose answer 1 or 2 for 1 point, answer 3 for 0 point), and add up the number of years of loneliness to obtain the cumulative burden score (ranged from 0 to 2 point; Figure 2).

The longitudinal changes in loneliness were divided into four groups by comparing the results of 2017 and 2018: never feeling lonely (always answer 3), persistent loneliness (answer 1 or 2 persistently), weakened loneliness (answer 1 or 2 becomes 3), and enhanced loneliness (answer 3 becomes 1 or 2).

### Covariates

Data on “age,” “sex,” “Urban and rural,” “educational level,” “marriage,” “living alone,” “exercise,” “subjective health,” “need for care,” “paid work,” “economic status,” “participation in public welfare,” “join geriatric society,” “non-spiritual cultural life,” “surf online,” “level of happiness,” and the number of chronic diseases, hypertension, diabetes, asthma, gastric disease, rheumatic disease, and malignant tumor were collected through the fourth SSAPUR questionnaire.

### Outcome

The study outcomes used the self-reported CCVD diagnosis by a physician in the 4th SSAPUR questionnaire to define CCVD, including angina pectoris, myocardial infarction, or stroke. Since the SSAPUR questionnaire is multi-thematic and not specifically designed to record CCVD, it may not emphasize other heart diseases such as heart failure or arrhythmias. In each year, participants were asked whether physicians had given them a diagnosis of cardiovascular disease (coronary heart disease/angina/stroke, etc.). The questionnaire has been well verified, used in CCVD surveys in several regions of China, and further verified by our internal research (18). All participants included in the analysis self-reported at baseline that they did not have CCVD, and the outcome was defined as the incidence of CCVD between 2018 and 2019.

### Statistics analysis

Categorical variables were described as numbers and proportions. The χ2 test was used to compare the differences among the categorical variables of baseline characteristics. A total of 105 covariates (1.7%) were missing, and no imputation was required.

To test the relationship between the cumulative burden of loneliness and new-onset CCVD, multivariate logistic proportional regression analysis was used to calculate odds ratios (ORs) with 95% confidence intervals (95% CIs). Three models were fitted: Model 1 was adjusted for age, sex, and residence. Model 2 was further adjusted for covariates in model 1 plus “educational level,” “marriage,” “living alone,” “exercise,” “subjective health,” “need for care,” “paid work,” “economic status,” “participation in public welfare,” “join geriatric society,” “non-spiritual cultural life,” “surf online,” and “level of happiness.” Model 3 was adjusted for covariates in model 2 plus the number of chronic diseases: hypertension, diabetes, asthma, gastric disease, rheumatic disease, and malignant tumor. For each comparison, any test results that reached the free statistical threshold of a value of p of <0.05 were then entered into the multiple linear regression model. Although some covariables had no significant differences at baseline, they were still adjusted in the model considering their correlation with the disease.

Subgroup analyses were performed to examine whether the association between the cumulative burden of loneliness and new-onset CCVD events was influenced by potential demographics and other covariates.

The following four sensitivity analyses were performed: (1) used cumulative burden scored by cumulative time to assess the impact on the risk of new-onset CCVD; (2) due to the possibility of reverse causation, we excluded participants with new-onset CCVD within the first year; (3) considering that older adult people living alone are more likely to form social isolation and have a mixed effect on loneliness, we excluded participants who lived alone; (4) we used the Markov Chain Monte Carlo (MCMC) multiple fill method (19) to supplement missing data.

A two-tailed value of *p* of <0.05 was considered to be statistically significant. All statistical analyses were carried out using SPSS24.0 (IBM Corp., Armonk, NY, United States).

## Results

### Demographic and sociological characteristics of baseline

A total of 2,562 people (27.9%) were lost to follow-up. The analysis included 6,181 participants (3,233 males (52.3%)) who completed the questionnaire and had clear loneliness and CCVD messages (Figure 1). The comparison of the baseline data between the included and lost follow-up subjects is presented in Supplementary Table S2. The population lost to follow-up was older, more male, from rural areas, had a lower educational level, a lower marriage rate, worse subjective health, fewer social activities, a lower sense of wellbeing, and a higher prevalence of malignant tumors. The general characteristics are listed in Supplementary Table S1. A total of 5,014 (81.1%) individuals had a lower cumulative burden of loneliness, and 1,167 (18.9%) individuals reported a higher level of cumulative burden. The overall prevalence of new-onset CCVD in 1 year was 11.5% and increased with the cumulative burden of loneliness (Table 1). With the cumulative burden of loneliness increasing, the proportion of female participants, living in the country, less educated, widowed, living alone, never exercising, having bad subjective health, needing care, having difficult economic status, not participating in social activities or spiritual culture, and feeling unhappy gradually increased (*p* < 0.001; Supplementary Table S1).

**Table 1:** Multivariate logistic analysis for new-onset CCVD.

Loneliness burden	*N* (*n* = 6,181)	Events (*n* = 710)	Incidence Rate	Model 1		Model 2		Model 3	
				OR(95%CI)	*p*-value	OR(95%CI)	*p*-value	OR(95%CI)	*p*-value
Cumulative burden									
Low	5,014	538	0.107	1 (reference)	-	1 (reference)	-	1 (reference)	-
High	1,167	172	0.147	1.396 (1.155–1.687)	0.001	1.363 (1.089–1.705)	0.007	1.373 (1.096–1.721)	0.006
Continuous variable									
Add 1 level	-	-	-	1.156 (1.067–1.252)	<0.001	1.147 (1.038–1.269)	0.007	1.141 (1.030–1.262)	0.011
Longitudinal changes in loneliness									
Never feel lonely	3,720	393	0.106	1 (reference)	-	1 (reference)	-	1 (reference)	-
persistent loneliness	811	115	0.142	1.348 (1.072–1.695)	0.011	1.300 (0.994–1.700)	0.055	1.301 (0.992–1.705)	0.057
Enhanced loneliness	834	99	0.119	1.115 (0.879–1.414)	0.370	1.058 (0.824–1.359)	0.658	1.020 (0.792–1.313)	0.879
weakened loneliness	816	103	0.126	1.188 (0.939–1.502)	0.151	1.167 (0.903–1.508)	0.239	1.159 (0.895–1.501)	0.264

Model 1 was adjusted for age, sex and residence. Model 2 was adjusted for covariates in model 1 plus “educational level” “marriage” “living alone” “exercise” “subjective health” “need for care” “paid work” “economic status” “participation in public welfare” “join geriatric society” “non-spiritual cultural life” “surf online” “level of happiness.” Model 3 was adjusted for covariates in model 2 plus number of chronic diseases, hypertension, diabetes, asthma, gastric disease, rheumatic disease, and malignant tumor.

### The impact of loneliness burden on new-onset CCVD

**Figure 3:**
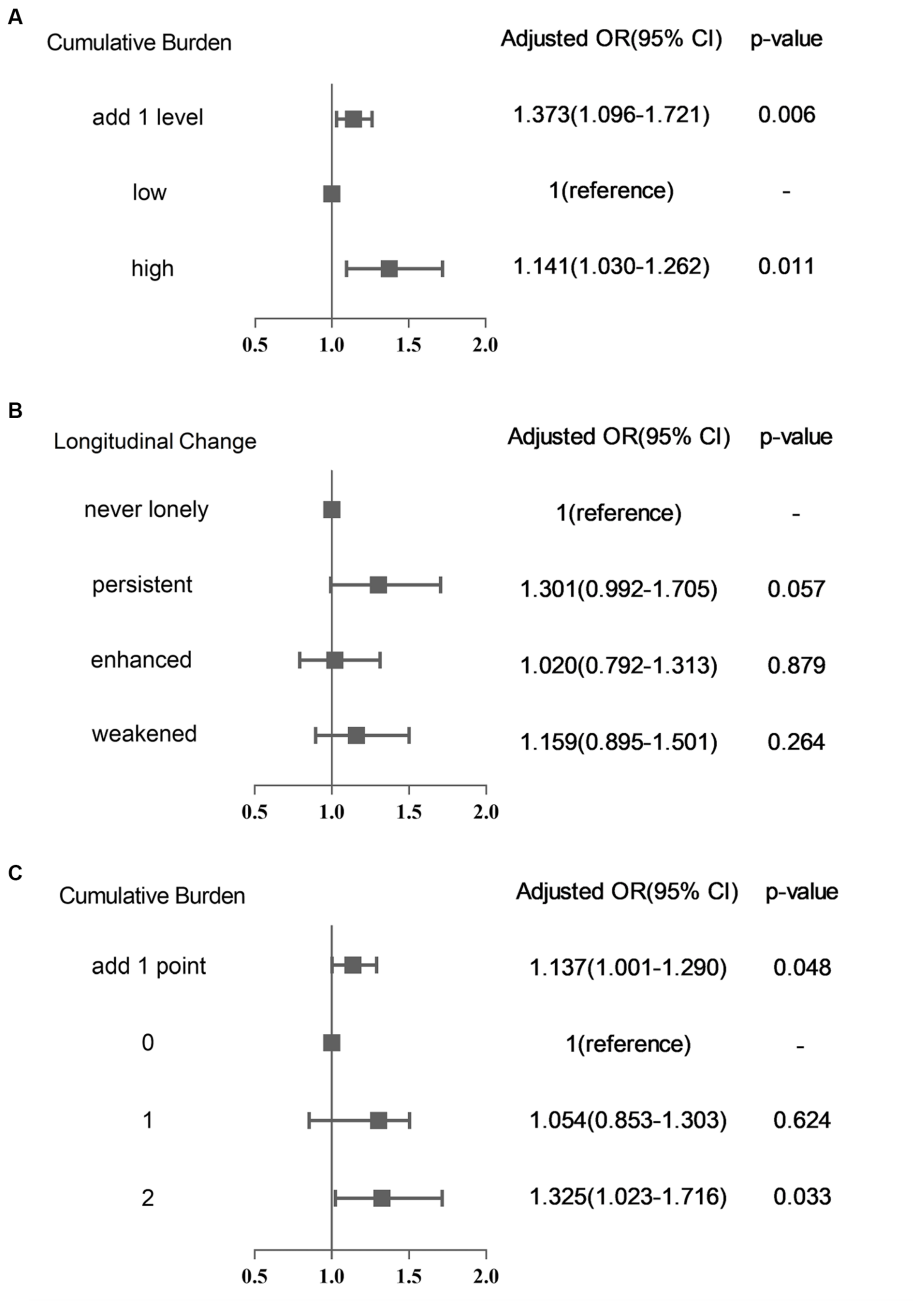
Compare the effect of different cumulative burdens and longitudinal changes in loneliness on the risk of the incidence of CCVD (All have been adjusted by Model 3). **(A)** Cumulative burden scored by cumulative degree; **(B)** compared with longitudinal change of loneliness; and **(C)** cumulative burden scored by cumulative time.

The OR and 95% CI between the cumulative burden of loneliness and new-onset CCVD risk are described in Table 1. Figure 3A shows the association between the cumulative burden scored by cumulative degree and the risk of developing CCVD events through adjustment (model 3). The risk of developing CCVD with a high cumulative burden was approximately 37% higher than in the low-burden group (OR 1.373, 95%CI 1.096–1.721; *p* = 0.006; Table 1).

In addition, we performed a multicollinearity test on the independent variables and found no collinearity problem between the covariates (Table 2).

**Table 2:** Multicollinearity test on the independent variables.

Factors	New-onset cardiac-cerebral vascular disease		
	β^b^	*p*-value	VIF
Cumulative burden Age	0.041 −0.014	0.005 0.317	1.343 1.216
Sex	−0.039	0.005	1.200
Education level	0.016	0.298	1.511
Marriage Living alone	−0.033 0.025	0.043 0.099	1.607 1.474
Exercise	−0.002	0.905	1.254
Health status	0.045	0.006	1.678
Require care	0.012	0.365	1.125
Gainful employment	0.010	0.465	1.100
Economic status	0.003	0.838	1.396
Not participating in public welfare activities	0.015	0.259	1.109
Join the geriatric society	0.002	0.888	1.066
Non-spiritual cultural life	0.007	0.592	1.120
Surf the Internet	−0.013	0.368	1.245
Happiness	−0.007	0.659	1.493
Urban and rural	0.007	0.632	1.169
Gastric disease	0.003	0.855	1.652
Asthma	−0.001	0.960	1.205
Diabetes	0.002	0.880	1.362
Hypertension	0.059	0.001	1.831
Malignant tumor	−0.005	0.674	1.036
Rheumatic disease	−0.045	0.013	2.024
Number of chronic diseases	0.064	0.020	4.659

β, parameter estimate.

### Longitudinal change in loneliness and risk of incident CCVD

Compared with never feeling lonely, persistent loneliness had a trend toward increased risk of CCVD, but the difference was not significant (OR 1.301, 95%CI:0.992–1.705; *p* = 0.057; Figure 3B). Both enhanced and weakened loneliness had potential trends to increase CCVD risk, but the effect was not significant as well (Table 1).

### Subgroup analyses

**Figure 4:**
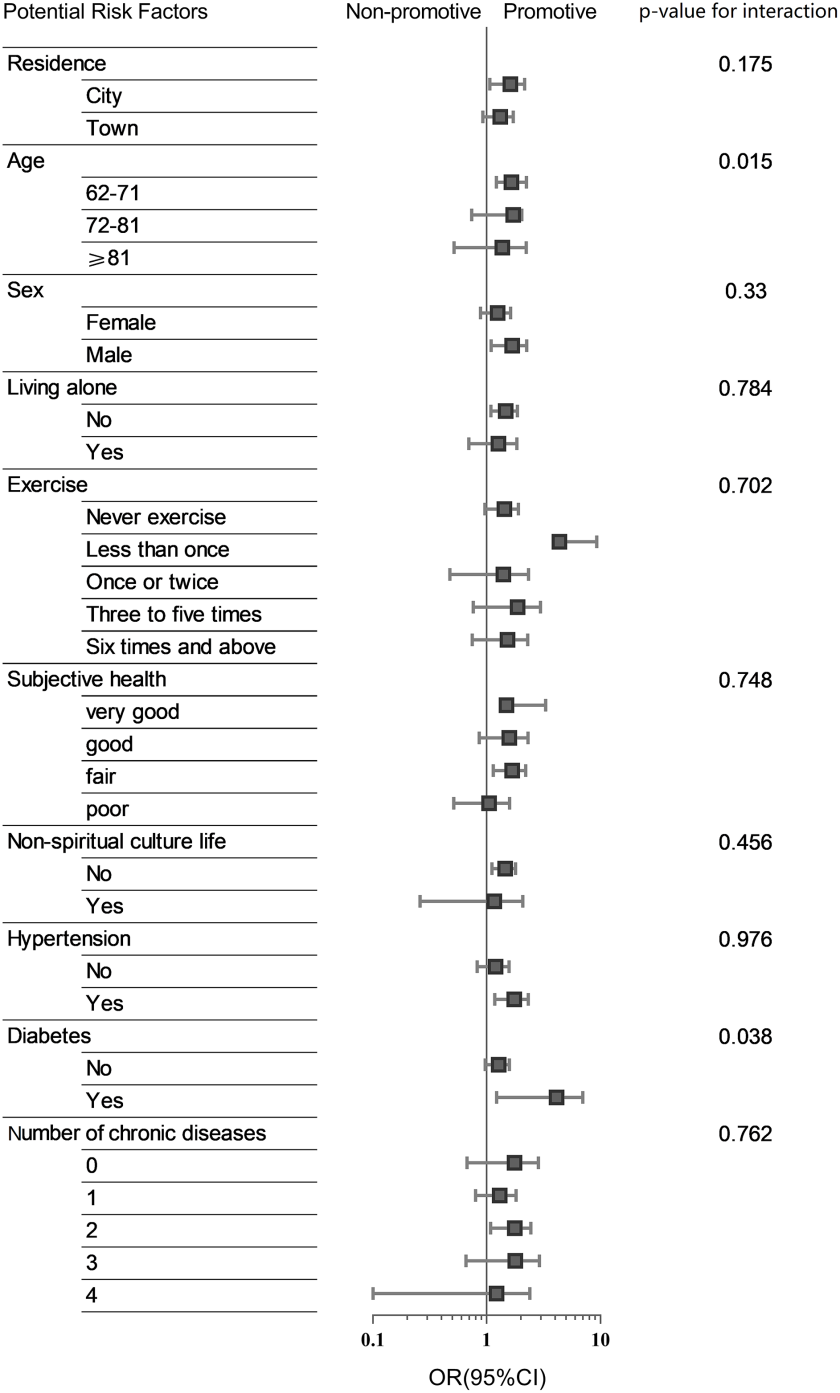
Subgroup analysis. Association between cumulative burden and a new-onset CCVD event stratified by part of potential risk factors.

Figure 4 shows the association between the cumulative burden of loneliness (scored by cumulative degree and calculated by the weighted method) and new-onset CCVD events stratified by underlying risk factors. There was no significant interaction between cumulative burden and risk factors, although male participants and subjects with hypertension who felt lonely were more likely to develop CCVD. However, the older adult with a loneliness burden between 62 and 71 years of age or with diabetes may have a higher risk of developing CCVD (*p* = 0.015 and *p* = 0.038 for interaction).

### Sensitivity analyses

Similar results were found when classifying the burden of loneliness based on the time of accumulation (Supplementary Table S3). After adjusting for confounding factors, being lonely for 2 consecutive years increased the risk of CCVD in the following year (OR 1.325, 95%CI 1.023–1.716; *p* = 0.033), and with each additional point of loneliness burden scored by cumulative time, the risk of developing CCVD increased by 13.7% (OR 1.137, 95%CI 1.001–1.290; *p* = 0.048) (Figure 3C). At the same time, the effect of the cumulative burden of loneliness on the risk of developing CCVD was still significant after excluding the first-year onset and solitary population (Supplementary Table S4). The results were also robust after multiple imputations.

## Discussion

The results of our study showed that the cumulative burden of loneliness had a predictive effect on the risk of CCVD in 1 year. After adjusting for covariates, including psychosocial factors and comorbidities, the risk of incidence of CCVD still increased with cumulative burden, regardless of whether they lived alone. However, the predictive effect of longitudinal change of loneliness on CCVD risk was not ideal, which may indicate that cumulative burden is more useful in predicting CCVD incidence by loneliness in the short term.

This study found that gender, place of residence, education, marriage, living alone, exercise, subjective health status, economic status, and so on are all important influencing factors of loneliness, which was the same as most studies (20, 21). Most studies have observed a link between poor social relationships and vascular diseases and clarified that loneliness suggests the possibility of an increased risk of CCVD (22–25). A nationally representative prospective cohort study from China (China Longitudinal Health and Retirement study) (11) also assessed the relationship between depressive symptoms and cardiovascular and cerebrovascular events in 12,417 middle-aged and older adult individuals without heart disease or stroke from 2011 to 2018. They found loneliness increased the risk of cardiovascular events by 21% (adjusted HR 1.21, 95%CI: 1.02–1.44). This study also concluded that loneliness was closely related to the risk of CCVD.

Numerous studies have confirmed that these adverse psychosocial factors are related to the morbidity and mortality of CCVD, and there may be a bidirectional causal relationship between loneliness and CCVD. This may explain some controversy regarding the impact of loneliness on CCVD. A study followed 479,054 people in the British biological database for 7.1 years and found that loneliness was associated with a higher risk of acute myocardial infarction (HR 1.49, 95%CI: 1.36–1.64) and stroke risk (HR 1.36, 95%CI: 1.20–1.55), but the correlation was significantly weakened after multiple factor adjustment (12). Other studies have shown that loneliness increases with age in older men, but no independent association has been found between loneliness and the risk of all-cause cardiovascular and non-cardiovascular death (13, 26). Some studies also suggested that loneliness is not as effective as social isolation, which can better reflect the risk of CCVD (27, 28). Our study found loneliness burden to be a potential risk factor for CCVD, which may indicate that loneliness is a regulated subjective feeling.

To evaluate the effect of this subjective feeling, we calculated the cumulative burden. Consistent with some research findings (29), our results found that the cumulative burden of different calculation methods reaching a certain score could predict the increased risk of CCVD. This can help with early prediction and timely intervention. Simultaneously, our study showed that longitudinal changes in loneliness were not predictive of the incidence of CCVD in the short term, which is inconsistent with other studies (30, 31). This difference may be related to the short follow-up time in this study; a single subjective change over a short period cannot accurately reflect the severity of the risk factor.

Most studies have shown that loneliness will increase with age. This study found that younger people suffering from loneliness had a greater impact on CCVD, which may be related to the fact that there were more older adult people in the lost follow-up population and there were more patients with malignant tumors, which may have resulted in death and loss of follow-up. Similar to other studies (32), we found that having diabetes was also a risk factor for CCVD in the older adult.

The effects of loneliness on CCVD differ across genders (33, 34). In the present study, we found fewer male participants with a higher cumulative burden of loneliness, which may also be related to more male participants being lost to follow-up. Previous studies have found a higher prevalence of CCVD in middle-aged and older adult men (35). Loneliness may act as a potential trigger for CCVD. Loneliness may reflect the imbalance between actual social relations and expectations. This imbalance will cause people to experience long-term stress challenges, which will become a possible mediator of psychological stress, leading to increased autonomic nervous activation, sympathetic-vagal imbalance, and the impairment of HPA axis regulation, which in turn creates the pathological basis of cardiovascular damage and leads to the occurrence of vascular obstructive disease (36). The mechanism of loneliness leading to organic CCVD is complex, and related studies have found that the increased risk of chronic disease and early death in lonely older adult people may also be associated with genetics (37) and cell aging (38). The relationship between the underlying mechanism and the cumulative burden of loneliness can be further investigated in the future.

This study had the following advantages: First, it was based on a nationally representative cohort with a large population and strong representation, which can better summarize the general situation of the social psychology of the Chinese older adult. Second, different calculations of cumulative burden helped a relatively consistent conclusion be obtained and reflected the clear impact of the cumulative effect of negative emotions on physical and mental health.

Our study has some limitations. First, we did not separately study the effect of loneliness on further classified CCVDs such as coronary heart disease, heart failure, stroke, or rheumatic heart disease. While some researchers have found that when there are multiple definitions of heart disease, respondents may choose the wrong classification, and self-reporting may be more effective at this time (26). Second, due to objective reasons, there was a lack of relevant investigations on smoking, alcohol consumption, and laboratory test indicators, such as cholesterol and low-density lipoprotein, which need further improvement in subsequent research. Moreover, self-reporting may be biased and lack a specific onset date for CCVD; thus, it was difficult to adjust for confounding factors that may occur in the outcome in a shorter period of time. Nevertheless, the applicability of the questionnaire has been proven in previous studies, as in other large-scale population surveys, and self-reported diseases have ensured a comparatively higher accuracy and authenticity (39). The study also lacked specific survival data, and the competing risks of death could not be assessed. To some extent, our study reflected the influence of loneliness severity on CCVD through a cumulative effect. Finally, the present study was also limited by its relatively short follow-up period, and further studies with longer tracking are still warranted.

## Conclusion

In summary, this national comprehensive cohort study showed that loneliness had a significant impact on CCVD among the older adult in China, and the cumulative burden of loneliness may be an indicator for assessing the risk of new-onset CCVD in the short term. As the cumulative burden increased, the predictive power for the occurrence of CCVD in the following year became stronger. We look forward to a longer-term follow-up to more accurately assess the role of loneliness burden. Regular psychological screening and intervention can help reduce health damage to the heart caused by psychological stress, thus reducing the burden of CCVD among the older adult in China and optimizing health management strategies for the older adult from the perspective of body–mind combination.

## Data availability statement

The original contributions presented in this study are included in the article/Supplementary material, further inquiries can be directed to the corresponding author.

## Ethics statement

The studies involving humans were approved by Ethics Review Committee of Beijing Hospital (2021BJYYEC-294-01). The studies were conducted in accordance with the local legislation and institutional requirements. The participants provided their written informed consent to participate in this study.

## Author contributions

DW: Formal analysis, Supervision, Visualization, Writing – original draft. XH: Conceptualization, Data curation. LM: Conceptualization, Data curation. JiL: Formal analysis, Software. JX: Formal analysis, Software. LZ: Formal analysis, Software. QM: Formal analysis, Software. HL: Investigation, Writing – review & editing. XZ: Investigation, Writing – review & editing. JuL: Methodology. QZ: Investigation, Writing – review & editing. DL: Funding acquisition, Writing – review & editing.

## Supplementary material

### Abbreviations

Abbreviation: CCVD, cardio-cerebral vascular disease.

## References

[1] Joseph P Leong D McKee M Anand SS Schwalm JD Teo K. Reducing the global burden of cardiovascular disease, part 1: the epidemiology and risk factors. Circ Res. (2017) 121:677–94. doi: 10.1161/CIRCRESAHA.117.308903 28860318

[2] Li X Wu C Lu J Chen B Li Y Yang Y. Cardiovascular risk factors in China: a nationwide population-based cohort study. Lancet Public Health. (2020) 5:e672–81. doi: 10.1016/S2468-2667(20)30191-2, PMID: 33271080

[3] Liu S Li Y Zeng X Wang H Yin P Wang L. Burden of cardiovascular diseases in China, 1990-2016: findings from the 2016 global burden of disease study. JAMA Cardiol. (2019) 4:342–52. doi: 10.1001/jamacardio.2019.0295, PMID: 30865215 PMC6484795

[4] Hamczyk MR del Campo L Andrés V. Aging in the cardiovascular system: lessons from Hutchinson-Gilford progeria syndrome. Annu Rev Physiol. (2018) 80:27–48. doi: 10.1146/annurev-physiol-021317-121454, PMID: 28934587

[5] Roth GA Johnson C Abajobir A Abd-Allah F Abera SF Abyu G. Global, regional, and National Burden of cardiovascular diseases for 10 causes, 1990 to 2015. J Am Coll Cardiol. (2017) 70:1–25. doi: 10.1016/j.jacc.2017.04.052, PMID: 28527533 PMC5491406

[6] Zhao D Liu J Wang M Zhang X Zhou M. Epidemiology of cardiovascular disease in China: current features and implications. Nat Rev Cardiol. (2019) 16:203–12. doi: 10.1038/s41569-018-0119-4, PMID: 30467329

[7] Hawkley LC Cacioppo JT. Loneliness matters: a theoretical and empirical review of consequences and mechanisms. Ann Behav Med. (2010) 40:218–27. doi: 10.1007/s12160-010-9210-8, PMID: 20652462 PMC3874845

[8] Hawkley LC Wroblewski K Kaiser T Luhmann M Schumm LP. Are U.S. older adults getting lonelier? Age, period, and cohort differences. Psychol Aging. (2019) 34:1144–57. doi: 10.1037/pag0000365, PMID: 31804118 PMC10621618

[9] Ong AD Uchino BN Wethington E. Loneliness and health in older adults: a Mini-review and synthesis. Gerontology. (2016) 62:443–9. doi: 10.1159/000441651, PMID: 26539997 PMC6162046

[10] Santini ZI Jose PE York Cornwell E Koyanagi A Nielsen L Hinrichsen C. Social disconnectedness, perceived isolation, and symptoms of depression and anxiety among older Americans (NSHAP): a longitudinal mediation analysis. Lancet Public Health. (2020) 5:e62–70. doi: 10.1016/S2468-2667(19)30230-0, PMID: 31910981

[11] Li H Zheng D Li Z Wu Z Feng W Cao X. Association of Depressive Symptoms with Incident Cardiovascular Diseases in middle-aged and older Chinese adults. JAMA Netw Open. (2019) 2:e1916591. doi: 10.1001/jamanetworkopen.2019.16591 31800066 PMC6902756

[12] Hakulinen C Pulkki-Råback L Virtanen M Jokela M Kivimäki M Elovainio M. Social isolation and loneliness as risk factors for myocardial infarction, stroke and mortality: UK biobank cohort study of 479 054 men and women. Heart. (2018) 104:1536–42. doi: 10.1136/heartjnl-2017-312663, PMID: 29588329

[13] Julsing JE Kromhout D Geleijnse JM Giltay EJ. Loneliness and all-cause, cardiovascular, and noncardiovascular mortality in older men: the Zutphen elderly study. Am J Geriatr Psychiatry. (2016) 24:475–84. doi: 10.1016/j.jagp.2016.01.136, PMID: 27066732

[14] Zeng X Jia N Meng L Shi J Li Y Hu X. A study on the prevalence and related factors of frailty and pre-frailty in the older population with diabetes in China: a national cross-sectional study. Front Public Health. (2022) 10:996190. doi: 10.3389/fpubh.2022.996190, PMID: 36211666 PMC9539138

[15] Hughes ME Waite LJ Hawkley LC Cacioppo JT. A short scale for measuring loneliness in large surveys: results from two population-based studies. Res Aging. (2004) 26:655–72. doi: 10.1177/0164027504268574, PMID: 18504506 PMC2394670

[16] Rafnsson SB Orrell M d'Orsi E Hogervorst E Steptoe A. Loneliness, social integration, and incident dementia over 6 years: prospective findings from the English longitudinal study of ageing. J Gerontol B Psychol Sci Soc Sci. (2020) 75:114–24. doi: 10.1093/geronb/gbx087, PMID: 28658937 PMC6909434

[17] Dai L Xu J Zhang Y Wang A Chen Z Mo J. Cumulative burden of lipid profiles predict future incidence of ischaemic stroke and residual risk. Stroke Vasc Neurol. (2021) 6:581–8. doi: 10.1136/svn-2020-000726, PMID: 33827914 PMC8717800

[18] Meng L Xu J Li J Hu J Xu H Wu D. Self-reported prevalence and potential factors influencing cardio-cerebral vascular disease among the Chinese elderly: a national cross-sectional study. Front Cardiovasc Med. (2022) 9:979015. doi: 10.3389/fcvm.2022.979015, PMID: 36337863 PMC9630358

[19] Enders CK. Multiple imputation as a flexible tool for missing data handling in clinical research. Behav Res Ther. (2017) 98:4–18. doi: 10.1016/j.brat.2016.11.008, PMID: 27890222

[20] Zuo S Lin L Chen S Wang Z Tian L Li H. Influencing factors of loneliness among older adults in China: a systematic review and meta-analysis. Psychogeriatrics. (2023) 23:164–76. doi: 10.1111/psyg.12897, PMID: 36270596

[21] Meehan DE Grunseit A Condie J HaGani N Merom D. Social-ecological factors influencing loneliness and social isolation in older people: a scoping review. BMC Geriatr. (2023) 23:726. doi: 10.1186/s12877-023-04418-8, PMID: 37946155 PMC10636946

[22] Stringhini S Zaninotto P Kumari M Kivimäki M Lassale C Batty GD. Socio-economic trajectories and cardiovascular disease mortality in older people: the English longitudinal study of ageing. Int J Epidemiol. (2018) 47:36–46. doi: 10.1093/ije/dyx106, PMID: 29040623 PMC5837467

[23] Foti SA Khambaty T Birnbaum-Weitzman O Arguelles W Penedo F Espinoza Giacinto RA. Loneliness, cardiovascular disease, and diabetes prevalence in the Hispanic community health study/study of Latinos sociocultural ancillary study. J Immigr Minor Health. (2020) 22:345–52. doi: 10.1007/s10903-019-00885-7 30963348 PMC6783350

[24] Christensen AV Juel K Ekholm O Thrysøe L Thorup CB Borregaard B. Significantly increased risk of all-cause mortality among cardiac patients feeling lonely. Heart. (2020) 106:140–6. doi: 10.1136/heartjnl-2019-315460, PMID: 31685646

[25] Barth J Schneider S von Känel R. Lack of social support in the etiology and the prognosis of coronary heart disease: a systematic review and meta-analysis. Psychosom Med. (2010) 72:229–38. doi: 10.1097/PSY.0b013e3181d01611, PMID: 20223926

[26] Hegeman A Schutter N Comijs H Holwerda T Dekker J Stek M. Loneliness and cardiovascular disease and the role of late-life depression. Int J Geriatr Psychiatry. (2018) 33:e65–72. doi: 10.1002/gps.4716, PMID: 28418079

[27] Yu B Steptoe A Chen LJ Chen YH Lin CH Ku PW. Social isolation, loneliness, and all-cause mortality in patients with cardiovascular disease: a 10-year follow-up study. Psychosom Med. (2020) 82:208–14. doi: 10.1097/PSY.0000000000000777, PMID: 31842061

[28] Bu F Zaninotto P Fancourt D. Longitudinal associations between loneliness, social isolation and cardiovascular events. Heart. (2020) 106:1394–9. doi: 10.1136/heartjnl-2020-316614, PMID: 32461329 PMC7497558

[29] Valtorta NK Kanaan M Gilbody S Hanratty B. Loneliness, social isolation and risk of cardiovascular disease in the English longitudinal study of ageing. Eur J Prev Cardiol. (2018) 25:1387–96. doi: 10.1177/2047487318792696, PMID: 30068233

[30] Vingeliene S Hiyoshi A Lentjes M Fall K Montgomery S. Longitudinal analysis of loneliness and inflammation at older ages: English longitudinal study of ageing. Psychoneuroendocrinology. (2019) 110:104421. doi: 10.1016/j.psyneuen.2019.104421 31494341

[31] Freak-Poli R Ryan J Neumann JT Tonkin A Reid CM Woods RL. Social isolation, social support and loneliness as predictors of cardiovascular disease incidence and mortality. BMC Geriatr. (2021) 21:711. doi: 10.1186/s12877-021-02602-2, PMID: 34922471 PMC8684069

[32] Wang X Ma H Li X Heianza Y Fonseca V Qi L. Joint association of loneliness and traditional risk factor control and incident cardiovascular disease in diabetes patients. Eur Heart J. (2023) 147:523. doi: 10.1161/circ.147.suppl_1.P523 PMC10361009 37385629

[33] Novak M Waern M Johansson L Zettergren A Ryden L Wetterberg H. Cardiovascular and all-cause mortality attributable to loneliness in older Swedish men and women. BMC Geriatr. (2020) 20:201. doi: 10.1186/s12877-020-01603-x, PMID: 32517656 PMC7285599

[34] Kraav SL Awoyemi O Junttila N Vornanen R Kauhanen J Toikko T. The effects of loneliness and social isolation on all-cause, injury, cancer, and CVD mortality in a cohort of middle-aged Finnish men. A prospective study. Aging Ment Health. (2021) 25:2219–28. doi: 10.1080/13607863.2020.1830945 33939562

[35] GBD 2016 Neurology Collaborators. Global, regional, and national burden of neurological disorders, 1990-2016: a systematic analysis for the global burden of disease study 2016. Lancet Neurol. (2019) 18:459–80. doi: 10.1016/S1474-4422(18)30499-X, PMID: 30879893 PMC6459001

[36] Siegrist J. Long-term stress in daily life in a socioepidemiologic perspective. Adv Psychosom Med. (2001) 22:91–103. doi: 10.1159/000059278 11477943

[37] Dennis J Sealock J Levinson RT Farber-Eger E Franco J Fong S. Genetic risk for major depressive disorder and loneliness in sex-specific associations with coronary artery disease. Mol Psychiatry. (2021) 26:4254–64. doi: 10.1038/s41380-019-0614-y, PMID: 31796895 PMC7266730

[38] Wilson SJ Woody A Padin AC Lin J Malarkey WB Kiecolt-Glaser JK. Loneliness and telomere length: immune and parasympathetic function in associations with accelerated aging. Ann Behav Med. (2019) 53:541–50. doi: 10.1093/abm/kay064, PMID: 30107521 PMC6499407

[39] Xie W Zheng F Yan L Zhong B. Cognitive decline before and after incident coronary events. J Am Coll Cardiol. (2019) 73:3041–50. doi: 10.1016/j.jacc.2019.04.019 31221251

