# Supplementary material for: Influence of loneliness burden on cardio-cerebral vascular disease among the Chinese older adult: a national cohort study
Source: Front Public Health. 2024 Feb 13;12:1307927. doi: 10.3389/fpubh.2024.1307927 (PMC10896831; doi:10.3389/fpubh.2024.1307927)
Supplement: Supplementary file 1 [file Table_1.DOCX]

**Supplementary Table 1. The based information of follow-up participants with different loneliness burden by weighted. (**scored by cumulative degree**)**

| Factor | Total (%)  (n=6181) | Cumulative burden of loneliness | | |
| --- | --- | --- | --- | --- |
|  |  | Low  (n=5014) (%) | High  (n=1167) (%) | P-value |
| Age（years） |  |  |  | <0.001 |
| 62-71 | 3815(61.7) | 3256(64.9) | 559(47.9) |  |
| 72-81 | 1796(29.1) | 1383(27.6) | 413(35.4) |  |
| ≥82 | 570(9.2) | 375(7.5) | 195(16.7) |  |
| Sex |  |  |  | <0.001 |
| Female | 2948(47.7) | 2289(45.7) | 659(56.5) |  |
| Male | 3233(52.3) | 2725(54.3) | 508(43.5) |  |
| Residence |  |  |  | <0.001 |
| City | 3067(49.6) | 2613(52.1) | 454(38.9) |  |
| Country | 3114(50.4) | 2401(47.9) | 713(61.1) |  |
| Education level |  |  |  | <0.001 |
| Have not attended school (including literacy classes) | 1587(25.7) | 1173(23.5) | 414(35.5) |  |
| Primary school (including private school) | 2794(45.3) | 2229(44.6) | 565(48.4) |  |
| Junior high school | 1188(19.3) | 1059(21.2) | 129(11.1) |  |
| High school/secondary school/vocational high school | 432(7.0) | 386(7.7) | 46(3.9) |  |
| College | 119(1.9) | 112(2.2) | 7(0.6) |  |
| Bachelor degree and above | 48(0.8) | 42(0.8) | 6(0.5) |  |
| Marriage |  |  |  | <0.001 |
| Married | 4507(73.5) | 4095(82.2) | 412(35.7) |  |
| Widowed | 1488(24.3) | 815(16.4) | 673(58.3) |  |
| Divorce | 42(0.7) | 26(0.5) | 15(1.4) |  |
| Never married | 99(1.6) | 46(0.9) | 53(4.6) |  |
| Living alone |  |  |  |  |
| No | 5197(84.2) | 4503(89.9) | 694(59.6) |  |
| Yes | 978(15.8) | 507(10.1) | 471(40.4)) |  |
| Exercise |  |  |  | <0.001 |
| Never exercise | 2539(41.1) | 1929(38.5) | 610(52.3) |  |
| Less than once | 292(4.7) | 229(4.6) | 63(5.4) |  |
| Once or twice | 823(13.3) | 659(13.2) | 164(14.1) |  |
| Three to five times | 867(14.0) | 708(14.1) | 159(13.6) |  |
| Six times and above | 1651(26.7) | 1480(29.6) | 171(14.7) |  |
| subjective health |  |  |  | <0.001 |
| Very good | 696(11.3) | 653(13.0) | 43(3.7) |  |
| Good | 2054(33.3) | 1781(35.6) | 273(23.4) |  |
| Fair | 2538(41.1) | 1995(39.9) | 543(46.6) |  |
| Poor | 788(12.8) | 522(10.4) | 266(22.8) |  |
| Very bad | 93(1.5) | 53(1.1) | 40(3.4) |  |
| Require care |  |  |  | <0.001 |
| No | 5588(90.4) | 4619(92.2) | 969(83.1) |  |
| Yes | 590(9.6) | 393(7.8) | 197(16.9) |  |
| Gainful employment |  |  |  | <0.001 |
| No | 5398(87.5) | 4311(86.2) | 1087(93.4) |  |
| Yes | 770(12.5) | 693(13.8) | 77(6.6) |  |
| Economic status |  |  |  | <0.001 |
| Very generous | 150(2.4) | 145(2.9) | 5(0.4) |  |
| Relatively ample | 1121(18.2) | 1015(20.3) | 106(9.1) |  |
| Basically enough | 3728(60.5) | 3088(61.8) | 640(54.9) |  |
| Tougher | 1000(16.2) | 665(11.3) | 335(28.7) |  |
| Very difficult | 167(2.7) | 87(1.7) | 80(6.9) |  |
| Not participating in public welfare activities |  |  |  | <0.001 |
| No | 3462(56.0) | 2901(57.9) | 561(48.1) |  |
| Yes | 2716(44.0) | 2111(42.1) | 605(51.9) |  |
| Join geriatric society |  |  |  | <0.001 |
| No | 5050(81.7) | 4043(80.7) | 1007(86.4) |  |
| Yes | 1128(18.3) | 969(19.3) | 159(13.6) |  |
| Non-spiritual cultural life |  |  |  | <0.001 |
| No | 5793(93.7) | 4776(95.3) | 1017(87.1) |  |
| Yes | 387(6.3) | 237(4.7) | 150(12.9) |  |
| Surf the Internet |  |  |  | <0.001 |
| No | 5713(92.4) | 4584(91.4) | 1129(96.7) |  |
| Yes | 468(7.6) | 439(8.6) | 38(3.3) |  |
| Feel happiness |  |  |  | <0.001 |
| Very | 1507(24.4) | 1436(28.6) | 71(6.1) |  |
| Relative | 2843(46.0) | 2442(48.7) | 401(34.4) |  |
| Common | 1596(25.8) | 1053(21.0) | 543(46.4) |  |
| Relative not | 194(3.1) | 68(1.4) | 126(10.8) |  |
| Very not | 40(0.6) | 14(0.3) | 26(2.2) |  |
| Hypertension |  |  |  | 0.801 |
| No | 4066(65.8) | 3302(65.9) | 765(65.5) |  |
| Yes | 2115(34.2) | 1712(34.1) | 403(34.5) |  |
| Gastric disease |  |  |  | <0.001 |
| No | 5191(84.0) | 4260(85.0) | 931(79.8) |  |
| Yes | 990(16.0) | 754(15.0) | 236(20.2) |  |
| Asthma |  |  |  | <0.001 |
| No | 5941(96.1) | 4845(96.6) | 1096(93.9) |  |
| Yes | 240(3.9) | 169(3.4) | 71(6.1) |  |
| Diabetes |  |  |  | 0.711 |
| No | 5668(91.7) | 4601(91.8) | 1067(91.4) |  |
| Yes | 513(8.3) | 413(8.2) | 100(8.6) |  |
| Rheumatic disease |  |  |  | <0.001 |
| No | 3782(61.2) | 3182(63.5) | 600(51.4) |  |
| Yes | 2399(38.8) | 1832(36.5) | 567(48.6) |  |
| Malignant tumor |  |  |  | 0.036 |
| No | 6136(99.3) | 4972(99.2) | 1164(99.7) |  |
| Yes | 45(0.7) | 42(0.8) | 3(0.3) |  |
| Number of chronic diseases |  |  |  | <0.001 |
| 0 | 1460(23.6) | 1288(25.7) | 172(14.7) |  |
| 1 | 2360(38.3) | 1900(37.9) | 460(39.4) |  |
| 2 | 1513(24.5) | 1187(23.7) | 326(27.9) |  |
| 3 | 612(9.9) | 468(9.3) | 144(12.3) |  |
| 4 | 236(3.8) | 171(3.4) | 65(5.6) |  |

**Supplementary Table 2.** The based information between participants of included and lost to follow-up.

| Factor | Lost to follow-up  (n=2569) (%) | Included  (n=6181) (%) | P-value |
| --- | --- | --- | --- |
| Age |  |  | <0.001 |
| 62-71y | 54.0 | 61.7 |  |
| 72-81 | 29.9 | 29.1 |  |
| ≥82 | 16.1 | 9.2 |  |
| Male | 55.0 | 52.3 | 0.021 |
| Country | 54.0 | 50.4 | 0.002 |
| Education level |  |  | 0.018 |
| Have not attended school (including literacy classes) | 28.7 | 25.7 |  |
| Primary school (including private school) | 42.2 | 45.3 |  |
| Junior high school | 18.9 | 19.3 |  |
| High school/secondary school/vocational high school | 6.8 | 7.0 |  |
| College | 2.5 | 1.9 |  |
| Bachelor degree and above | 0.9 | 0.8 |  |
| Marriage |  |  | 0.004 |
| Married | 69.7 | 73.5 |  |
| Widowed | 27.4 | 24.3 |  |
| Divorce | 0.9 | 0.7 |  |
| Never married | 2.0 | 1.6 |  |
| Living alone | 15.9 | 15.8 | 0.918 |
| Exercise |  |  | 0.057 |
| Never exercise | 43.0 | 41.1 |  |
| Less than once | 4.6 | 4.7 |  |
| Once or twice | 12.9 | 13.3 |  |
| Three to five times | 15.4 | 14.0 |  |
| Six times and above | 24.1 | 26.7 |  |
| Subjective health |  |  | <0.001 |
| Very good | 8.9 | 11.3 |  |
| Good | 29.8 | 33.3 |  |
| Fair | 42.2 | 41.1 |  |
| Poor | 15.5 | 12.8 |  |
| Very bad | 3.5 | 1.5 |  |
| Require care | 19.4 | 9.6 | <0.001 |
| Have gainful employment | 9.4 | 12.5 | <0.001 |
| Economic status |  |  |  |
| Very generous | 2.0 | 2.4 |  |
| Relatively ample | 19.2 | 18.2 |  |
| Basically enough | 59.4 | 60.5 |  |
| Tougher | 16.5 | 16.2 |  |
| Very difficult | 2.8 | 2.7 |  |
| Not participating in public welfare activities | 54.0 | 44.4 | <0.001 |
| Join geriatric society | 17.6 | 18.3 | 0.436 |
| Non-spiritual cultural life | 8.9 | 6.3 | <0.001 |
| Surf the Internet | 7.6 | 7.6 | 0.926 |
| Feel happiness |  |  | <0.001 |
| Very | 18.7 | 24.4 |  |
| Relative | 47.8 | 46.0 |  |
| Common | 29.8 | 25.8 |  |
| Relative not | 3.2 | 3.1 |  |
| Very not | 0.6 | 0.6 |  |
| Hypertension | 35.5 | 34.2 | 0.237 |
| Gastric disease | 14.8 | 16.0 | 0.138 |
| Asthma | 4.3 | 3.9 | 0.342 |
| Diabetes | 7.5 | 8.3 | 0.218 |
| Rheumatic disease | 36.4 | 38.8 | 0.034 |
| Malignant tumor | 1.6 | 0.7 | <0.001 |
| Number of chronic diseases |  |  | 0.628 |
| 0 | 24.4 | 25.4 |  |
| 1 | 39.4 | 37.8 |  |
| 2 | 23.6 | 23.8 |  |
| 3 | 9.3 | 9.4 |  |
| 4 | 3.3 | 3.6 |  |

**Supplementary Table 3.** Multivariate logistic analysis for new-onset CCVD (scored by cumulative time).

| Loneliness burden | N  (n=6184) | Events  (n=710) | Incidence Rate | Model 1 | | Model 2 | | Model 3 | |
| --- | --- | --- | --- | --- | --- | --- | --- | --- | --- |
|  |  |  |  | OR (95%CI) | P-value | OR(95%CI） | P-value | OR(95%CI） | P-value |
| continuous variable | | | | | | | | | |
| Add 1 point | - | - | - | 1.168(1.055-1.294) | 0.003 | 1.144(1.009-1.297) | 0.036 | 1.137(1.001-1.290) | 0.048 |
| categorical variables | | | | | | | | | |
| 0 | 3720 | 393 | 0.106 | 1(reference) | - | 1(reference) | - | 1(reference) | - |
| 1 | 1419 | 165 | 0.116 | 1.091(0.897-1.328) | 0.381 | 1.078(0.874-1.329) | 0.485 | 1.054(0.853-1.303) | 0.624 |
| 2 | 1042 | 152 | 0.146 | 1.393(1.313-1.715) | 0.002 | 1.335(1.033-1.726) | 0.027 | 1.325(1.023-1.716) | 0.033 |

Model 1 was adjusted for age, sex and residence.

Model 2 was adjusted for covariates in model 1 plus “educational level” “marriage” “living alone” “exercise” “subjective health” “need for care” “paid work” “economic status” “participation in public welfare” “join geriatric society” “non-spiritual cultural life” “surf online” “level of happiness”.

Model 3 was adjusted for covariates in model 2 plus number of chronic diseases, hypertension, diabetes, asthma, gastric disease, rheumatic disease, malignant tumor.

**Supplementary Table 4**. Additional sensitivity analysis verifies the impact of cumulative burden of loneliness on the risk of CCVD onset.

| Loneliness burden | N  (n=5765) | Events  (n=710) | Incidence Rate | Model 1 | | Model 2 | | Model 3 | |
| --- | --- | --- | --- | --- | --- | --- | --- | --- | --- |
|  |  |  |  | OR(95%CI） | P-value | OR(95%CI） | P-value | OR(95%CI） | P-value |
| Cumulative Burden (Exclude new-onset CCVD in the first year) | | | | | | | | | |
| Low | 4676 | 538 | 0.115 | 1(reference) | - | 1(reference) | - | 1(reference) | - |
| High | 1089 | 172 | 0.158 | 1.395(1.153-1.688) | 0.001 | 1.338(1.067-1.678) | 0.012 | 1.350(1.074-1.697) | 0.010 |
| Cumulative Burden (Exclude living alone at baseline) | | | | | | | | | |
| Low | 4503 | 478 | 0.106 | 1(reference) | - | 1(reference) | - | 1(reference) | - |
| High | 694 | 102 | 0.147 | 1.381(1.091-1.748) | 0.007 | 1.439(1.108-1.868) | 0.006 | 1.437(1.104-1.869) | 0.007 |
| Cumulative Burden (After multiple imputation) | | | | | | | | | |
| Low | 5014 | 538 | 0.107 | 1(reference) | - | 1(reference) | - | 1(reference) | - |
| High | 1167 | 172 | 0.147 | 1.396(1.155-1.687) | 0.001 | 1.331(1.066-1.661) | 0.012 | 1.342(1.073-1.677) | 0.010 |

Model 1 was adjusted for age, sex and residence.

Model 2 was adjusted for covariates in model 1 plus “educational level” “marriage” “living alone” “exercise” “subjective health” “need for care” “paid work” “economic status” “participation in public welfare” “join geriatric society” “non-spiritual cultural life” “surf online” “level of happiness”.

Model 3 was adjusted for covariates in model 2 plus number of chronic diseases, hypertension, diabetes, asthma, gastric disease, rheumatic disease, malignant tumor.
